# Longitudinal Assessment of Pulmonary Function and Bronchodilator Response in Pediatric Patients With Post-infectious Bronchiolitis Obliterans

**DOI:** 10.3389/fped.2021.674310

**Published:** 2021-05-19

**Authors:** Xiuhua Yu, Jiaoyang Wei, Yanchun Li, Lu Zhang, Hongming Che, Li Liu

**Affiliations:** ^1^Department of Pediatrics, The First Hospital of Jilin University, Changchun, China; ^2^Department of Pediatrics, The Hospital of Shandong Technology and Business University, Yantai, China

**Keywords:** postinfectious bronchiolitis obliterans, children, pulmonary function, bronchodilator response, longitudinal assessment

## Abstract

**Backgroud:** Postinfectious bronchiolitis obliterans (PIBO) is a rare respiratory disease. In recent years, the disease has been recognized and diagnosed increasingly in children. Pulmonary function is important for diagnosis, identifying the severity of the PIBO and monitoring progression. But there have been only a few studies that followed the evolution of PIBO on the basis of pulmonary function tests (PFTs).

**Objective:** The study targeted the evolution of pulmonary function and bronchodilator response in a case series of Chinese children with PIBO.

**Methods:** Twelve children between the ages of 6–99 months with PIBO were studied retrospectively from 2009 to 2019. Forced vital capacity (FVC), forced expiratory volume in 1 s (FEV_1_), the FEV_1_/FVC ratio, and maximal midexpiratory flow velocity 25–75% (MMEF_25−75%)_ were collected at each PFT, and bronchodilator responses were evaluated. Spirometric parameters were monitored over time, and generalized linear mixed models were used to analyze longitudinal panel data.

**Results:** The median baseline PFT values for FVC, FEV_1_, the FEV_1_/FVC ratio, and MMEF_25−75%_ were 41.6, 39.75, 90.7, and 22.2%, respectively. At the initial PFTs, 10 (83.3%) patients demonstrated a significant bronchodilator response. FVC and FEV_1_ increased by 8.212%/year and 5.007%/year, respectively, and the FEV_1_/FVC ratio decreased by an average of 3.537%/year. MMEF_25−75%_ showed improvement at an average rate of 1.583% every year. Overall, FEV_1_ and MMEF_25−75%_ showed different degrees of improvement after the use of inhaled bronchodilators at each PFT session for 10 patients, and FEV_1_ measures demonstrated significant (>12%) β_2_-bronchodilation in 56% of PFT sessions.

**Conclusions:** Pediatric patients with PIBO showed an obstructive defect in pulmonary function. The FVC, FEV_1_, and MMEF_25−75%_ improved as they grew older, while the FEV_1_/FVC ratio decreased. This may be due to the development of lung parenchyma more than airway growth. Airway obstruction in some patients improved with the use of β_2_ agonists.

## Introduction

Bronchiolitis obliterans (BO) refers to a rarely seen small airway injury–related chronic inflammatory airflow obstruction syndrome. Many conditions may trigger BO, such as infection, lung transplantation, bone marrow transplantation, exposure to toxic gases, chronic aspiration, connective tissue diseases, and certain drugs (1). Postinfectious BO (PIBO) is especially common in children. Some studies reported that PIBO was secondary to the infectious incidence of adenovirus, influenza, parainfluenza, respiratory syncytial virus (RSV), measles virus, *Mycoplasma pneumoniae* and others (2–4). The histopathological characteristics of PIBO are concentric narrowing and obliteration of small airways as a result of an inflammatory process of the bronchiolar lumen (5). Its primary clinical manifestations are usually repeated cough, wheezing and shortness of breath, accompanied by varying degrees of dyspnea, and decreased activity tolerance. In addition to clinical characteristics, pulmonary function demonstrates severe airway obstruction, and high-resolution computed tomography (HRCT) shows mosaic perfusion patterns and bronchiectasis (2, 3). Some scholars have proposed a PIBO score to diagnose the disease. The presenting clinical history is scored as four points, adenovirus infection is scored as three points, and chest HRCT with a mosaic perfusion pattern is scored as four points. A score over seven can be a predictor of the diagnosis (6).

In PIBO, pulmonary function is important for diagnosis, identifying the severity of the illness and monitoring progression. PIBO usually occurs in infants (4), who cannot perform the spirometry maneuver, and patients are sometimes lost during the follow-up periods; therefore, there have been only a few studies that followed the evolution of PIBO on the basis of pulmonary function tests (PFTs) (7–10). Conversely, PIBO is not a common disease, and although it was first described in 1901, the disease has been infrequently recognized and diagnosed. Our current knowledge about the evolution of pulmonary function in children with PIBO is limited and subject to controversy. PIBO is usually considered a disorder involving fixed obstruction with no significant bronchodilator response. Some authors observed that pulmonary function in PIBO patients was unchanged and even declined with growth (8, 9). However, other studies demonstrated that lung function slowly improved (7, 10), and it has been reported that some patients with PIBO showed positive responses to β_2_ agonists (8, 11, 12). This research aims to assess the evolution of pulmonary function and bronchodilator response in a case series of Chinese children with PIBO. Profiling the longitudinal pulmonary function of children with PIBO was beneficial to the research and treatment of the disease.

## Methods

We conducted a retrospective analysis of the clinical data, PFT results, and HRCT features of patients with a diagnosis of PIBO between 2009 and 2019 at the First Hospital of Jilin University. All parts of the experiments constituted functional components of the clinical care of children with PIBO. This research was approved by the ethics committees of our research institutions. All participant patients and their parents or legal guardians agreed to research inclusion.

### Participants

Twelve pediatric patients were enrolled, and data were traced from electronic clinically recorded data. The diagnosis of PIBO was made on the basis of a typically traced serious lower respiratory tract infection or acute lung injury in children who were formerly in good condition; exercise intolerance, recurrent, or persistent wheezing, coughing and tachypnea with extensive wheezing and moist crackles in the lungs lasting for more than 6 weeks; severe obstructive lung disease on PFTs; and changes in the mosaic perfusion pattern, bronchial wall thickening, bronchial dilation, atelectasis, and vascular attenuation on chest HRCT (the median age at first HRCT was 32 months) (6). During the whole follow-up period, the HRCT findings were persistent (the median time interval between each HRCT was 9 months), and the symptoms could not be completely resolved even after treatment. Diagnoses of asthma, pulmonary tuberculosis, cystic fibrosis, bronchopulmonary dysplasia, congenital heart trouble, and immunodeficiency were excluded according to clinical, radiological and laboratory records. Allergy tests, including skin prick testing and measuring the level of specific immunoglobulin E in serum, were performed in all patients, and there was no positive finding. Bronchoscopy was also performed in five children to exclude other lung diseases. Direct immunofluorescence viral antigen testing of nasopharyngeal secretions was completed to identify adenovirus, influenza, RSV, and parainfluenza virus. Polymerase chain reaction (PCR) was performed to test *M. pneumoniae* and *Chlamydia pneumoniae*. Serum IgM testing was used to determine measles virus, sputum and serum culture were conducted to exclude bacteria infection. We followed patients' PFT results as soon as they were able to perform the spirometry maneuver until November 2019. The average time was 29 months (range of 11–48 months) for follow-up of PFTs. Ten patients received no special therapy during the PFT analysis unless an acute respiratory tract infection was present, and two children were under intermittent low-dose inhaled corticosteroid (ICS) treatment (budesonide or fluticasone). However, ICS, oral low-dose corticosteroids (OCS, prednisone, 1–2 mg/kg.d, the quantity of medicine stepped dorm gradually after the first month, and stopped in 3–6 months) and azithromycin (5 mg/kg.d, 3 days per week, 3–6 months) were applied in all patients in the first year of PIBO diagnosis. Drugs may need to be used again if the condition worsened after an acute respiratory infection.

### PFT

Spirometry was performed in the pulmonary function laboratory through Jaeger Master Screen Paed (Jaeger Company, Wurzburg, Germany) by a trained physician and following American Thoracic Society (ATS)/European Respiratory Society (ERS) performance criteria to ensure accurate and reproducible results (13).

Actual flows (FEV_1_ in L; maximum mid-expiratory flow 25–75%, MMEF_25−75%_ in L/s), lung volumes (FVC, in L) and FEV_1_/FVC ratios were standardized for differences in height, age, and sex so that they could be converted to the predicted percentages according to Global Lung Function Initiative (GLI) reference equations. The initial PFT data from each patient were obtained when they could perform the maneuver, and the median age was 73.5 months (interquartile ranges, IQR: 69–87 months). The average follow-up time of PFTs was ~29 months (IQR: 11–48 months). PFTs were performed when pediatric patients had been clinically stable for at least 2 weeks, in the same place, using the same device and by the same physician. The mouth seal around the mouthpiece, breathing patterns and even body position were carefully assessed. Prior to the PFTs, long- and short-acting β_2_ agonists were withheld for 48 and 12 h, respectively. In accordance with the ATS/ERS suggestions, except for the baseline measurement, the PFT parameters were measured within 15 min after inhalation of 0.2% salbutamol solution (<6 years old, 1.25 ml, 6–12 years old, 1.875 ml, >12 years old, 2.5 ml) by a compression atomizer pump (Germany, PARI). The approaches to assessing bronchodilator reactions are displayed in **Chart 1**. In addition, the initial PFTs offer an analysis of the factors that may have affected bronchodilator response.

**Chart 1**. Different approaches to calculating a bronchodilator response.
Percent range out of the former (pre-bronchodilator) measurement:
  $$\left({\text{FEV}}_{1}\text{post}-{\text{FEV}}_{1}\text{pre}\right)\text{/}\left({\text{FEV}}_{1}\text{pre}\times 100\right)$$
Absolute volume range from the previous (pre-bronchodilator) measurement:
  $${\text{FEV}}_{1}\text{post}-{\text{FEV}}_{1}\text{pre}$$
Post: post-bronchodilator; and pre: pre-bronchodilator. MMEF_25−75%_ featured similarity

### Statistical Analysis

We used a longitudinal data analysis to assess the change in PFTs in past research. Quantitative data are expressed as medians and interquartile ranges, whereas, qualitative data are described by frequencies (composition ratios). Generalized linear mixed models were estimated based on age at PFTs as the fixed effects, and random effects were given special presentation at the level of the individual effects. The decreases and increases in PFT parameters were expressed in relation to changes in the partial percentages of predicted values for height, age, and sex. At the initial PFTs, a linear mixed model was employed to determine whether bronchodilator reactions were influenced by age at the time of diagnosis or by allergy-related factors (including a trace of wheezing, atopic dermatitis and family asthma history). The analysis of the complete data was performed using R software version 3.5.3 (Auckland, New Zealand). A *p*-value <0.05 was considered statistically significant.

## Result

### Clinical Characteristics

**Figure 1:**
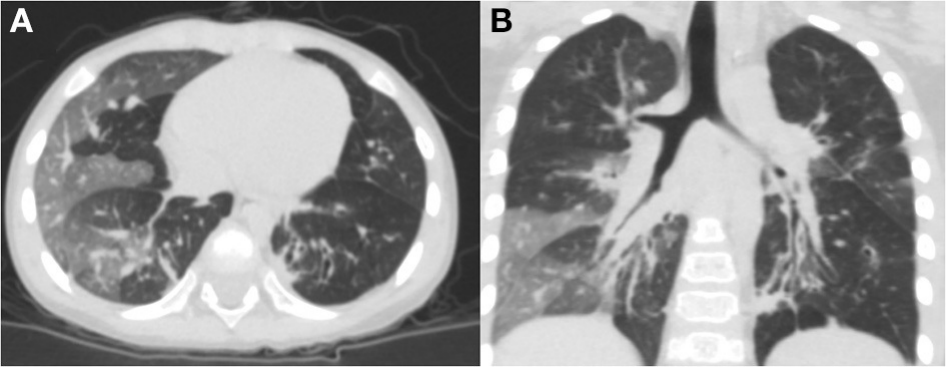
Chest HRCT scans of a 2-year-old boy with PIBO. **(A)** Mosaic perfusion pattern. **(B)** Bronchial wall thickening and bronchial dilation (3D reconstruction).

The average age at diagnosis was 36 months for all previously healthy patients, and the median age at initial pulmonary injury was 26 months. There were eight boys (67%) and four girls (33%). The race/ethnicity of all patients was Han Chinese. During the initial severe lower respiratory infection, four patients were positive for adenovirus antigen as shown by testing nasopharyngeal secretions; *M. pneumoniae* was identified in four individuals; measles virus was found in one child with serum IgM antibody tests; and the infection etiology was unknown in two patients. In addition, one patient was infected with both adenovirus and *M. pneumoniae* (Table 1). Six patients required mechanical ventilation for the initial severe infection, and others needed oxygen supplementation. The diagnosis of PIBO was conducted according to clinical and HRCT findings. The symptoms of patients included the presentation of dyspnea, wheezing, cough, exercise intolerance, and frequent respiratory disease. Persistent moist crackles, extensive wheezing and some signs of hypoxemia (cyanosis, nares flaring, tachypnea, adjunctive respiratory muscle participation) were found on physical examination. However, during the PFT follow-up period, the symptoms improved to an extent in some children with PIBO. In addition, one patient needed intermittent home-oxygen administration in the first 2 years. In all 12 children, mosaic perfusion patterns, air trapping and bronchial wall thickening were observed on HRCT (the median duration of the disease at the time of first HRCT was 2 months). In one patient, bronchial dilation and atelectasis were observed. Bronchiectasis was also observed in another patient, while right atelectasis and thoracic collapse were observed in one 7-year-old boy (Figures 1A,B).

**Table 1:** Characteristics of the study subjects.

**Date of birth**	**Age at the time of diagnosis**	**Sex**	**Race**	**Etiology**	**Needed oxygen supplementation or mechanical ventilation at initial pulmonary injury**	**Allergy factors (including a history of wheezing, atopic dermatitis and family asthma history)**
25 September 2003	84 months	Male	Han	Measles virus	Mechanical ventilation	No
21 September 2009	15 months	Male	Han	Adenovirus	Mechanical ventilation	No
31 May 2011	14 months	Male	Han	Adenovirus	Mechanical ventilation	Eczema
12 December 2007	47 months	Male	Han	*M. pneumoniae*	Oxygen supplementation	No
18 August 2008	14 months	Male	Han	Adenovirus	Oxygen supplementation	Eczema Wheezing
29 December 2011	31 months	Male	Han	*M. pneumoniae*	Oxygen supplementation	No
12 December 2011	6 months	Male	Han	Unknown	Mechanical ventilation	No
17 July 2012	20 months	Female	Han	Adenovirus	Oxygen supplementation	Eczema
11 June 2010	24 months	Female	Han	*M. pneumoniae*	Oxygen supplementation	Eczema
17 August 2007	57 months	Female	Han	Adenovirus *M. pneumoniae*	Mechanical ventilation	No
6 November 2009	13 months	Female	Han	Unknown	Mechanical ventilation	Eczema Wheezing
26 February 2009	99 months	Male	Han	*M. pneumoniae*	Oxygen supplementation	Wheezing

### PFT

#### Baseline PFTs

Initial PFTs were performed when each child could complete the spirometry tests (median: 73.5 months; IQR: 69–87 months) according to the guidelines established by the ATS/ERS. The median time of initial PFT and PIBO diagnosis was 45 months. All PFT data indicated moderate to severe obstruction. At the baseline PFT, the median values (described as a percentage of the predicted values adjusted for height, age, and sex) for FEV_1_, FVC, the FEV_1_/FVC ratio, and MMEF_25−75%_ were 39.75, 41.6, 90.7, and 22.2%, respectively (Table 2).

**Table 2:** Baseline and final PFT values.

	**PFT values (median)**		**Change per year**	**95% CI**	**t**	***P*-value**
	**Baseline**	**End of study**				
FEV_1_	39.75%	57.8%	5.007%	3.463 to 6.552	6.356	<0.0001
FVC	41.6%	76.1%	8.212%	6.531 to 9.894	9.574	<0.0001
FEV_1_/FVC	90.7%	77.85%	−3.537%	−5.09 to −1.984	−4.464	<0.0001
MMEF_25−75%_	22.2%	26.2%	1.583%	0.046 to 3.12	2.019	0.048

*PFT, pulmonary function test; FVC, forced vital capacity; FEV1, forced expiratory volume in 1 s; MMEF_25−75%_, maximal midexpiratory flow velocity 25_−_75%; CI, confidence interval*.

The improvements in expiratory flows were significant after inhalation of bronchodilators, although, they did not reach normal predicted levels. According to the recommendation of ATS/ERS, when the cutoff point of FEV_1_ improvement as a percent change of 12% was used, 10 (83.3%) children with PIBO showed a significant bronchodilator response (data not shown). As shown in Table 3, FEV_1_ was significantly improved after inhalation of bronchodilators, as was MMEF_25−75%_. The median percent variation and the median absolute volume range based on the former measurements were 15.55% and 0.15 L for FEV_1_ and 49.4% and 0.24 L/s for MMEF_25−75%_, respectively. In the multivariate analysis on the outcome variables, our study found that the predictor variables (age at diagnosis, allergy factors) exerted no dramatic effects upon the bronchodilator reaction, although an older age at diagnosis reduced the improvement value of MMEF_25−75%_ (*P* < 0.05) (Table 4).

**Table 3:** Bronchodilator responses in pediatric patients with PIBO, considering the mean percent variation, and the mean absolute volume change from the previous measurement^*^.

**Variable**	**BD response**	
	**Baseline**	**End of study**
FEV_1_ (% change from previous)	15.55 (12.4–38.8)	16 (1–32)
MMEF_25−75%_ (% change from previous)	49.4 (27–62.7)	22.5 (9.1–40.7)
FEV_1_ (absolute volume change in L)	0.15 (0.09–0.18)	0.12 (0.02–0.31)
MMEF_25−75%_ (absolute volume change in L/s)	0.24 (0.09–0.47)	0.11 (0.05–0.25)

*PIBO, post-infectious bronchiolitis obliterans; BD, bronchodilator*.

* *Values described as median (interquartile range, IQR)*.

**Table 4:** Analysis of factors with a potential influence on bronchodilator responses in children with PIBO at the initial PFTs.

	**BETA**	**95% CI**	**t**	***P***
FEV_1_ improvement rate (BD response)				
Age at the time of diagnosis	0.135	−0.194~0.464	0.804	0.442
Allergy factors	19.915	0.669~39.161	2.028	0.073
MMEF_25−75%_ improvement rate (BD response)				
Age at the time of diagnosis	−0.255	−0.752~0.241	−1.007	0.340
Allergy factors	31.552	2.507~60.598	2.129	0.062
FEV_1_ improvement value (BD response)				
Age at the time of diagnosis	0.000	−0.001~0.002	0.229	0.824
Allergy factors	0.059	−0.038~0.156	1.183	0.267
MMEF_25−75%_ improvement value (BD response)				
Age at the time of diagnosis	−0.004	−0.008~−0.001	−2.294	0.047
Allergy factors	−0.010	−0.213~0.193	−0.097	0.924

*PIBO, post-infectious bronchiolitis obliterans; PFT, pulmonary function test; BD, bronchodilator*.

#### Final PFTs

The median duration of the PFT follow-up was 29 months (IQR: 11–48 months). At the final PFTs, the median values for FEV_1_, FVC, the FEV_1_/FVC ratio, and MMEF_25−75%_ were 57.8, 76.1, 77.85, and 26.2%, respectively (Table 2); seven (58.3%) children had positive results for bronchodilator response (data not shown). The improvements were also significant when the median percent variation was calculated (increases of 16 and 22.5% for FEV_1_ and MMEF_25−75%_ after inhalation of bronchodilators, respectively). In addition, the median absolute volume change was 0.12 L for FEV_1_ and 0.11 L/s for MMEF_25−75%_ (Table 3).

#### Progression of PFTs

**Figure 2:**
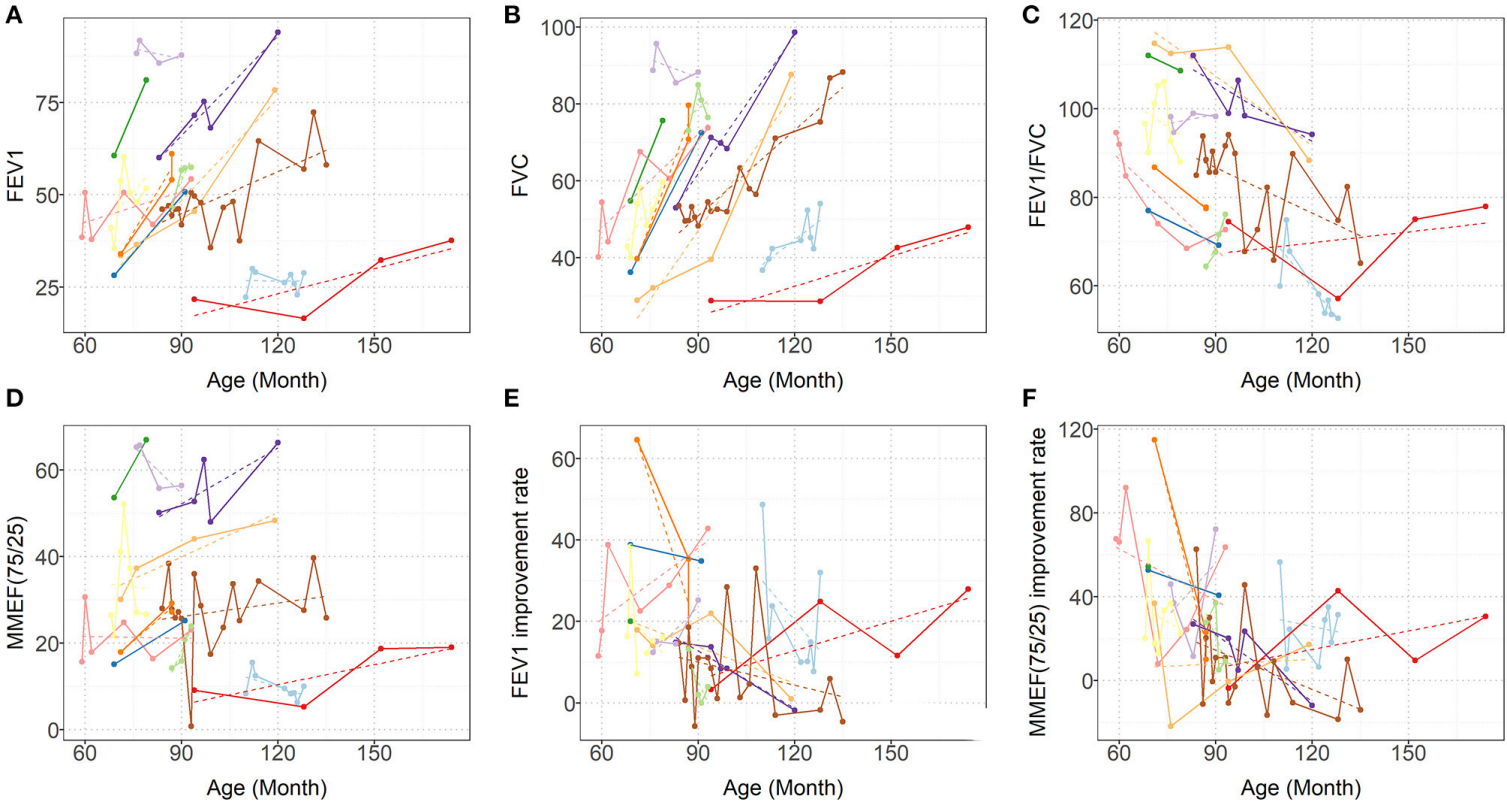
Predicted (dashed lines) and Observed (solid lines) progression of **(A)** FEV_1_, **(B)** FVC, **(C)** FEV_1_/FVC, **(D)** MMEF_25−75%_ **(E)** FEV_1_ improvement rate (BD response), and **(F)** MMEF_25−75%_ improvement rate (BD response). Each colored line represents one patient. FVC, forced vital capacity; FEV1, forced expiratory volume in 1 s; and MMEF_25−75%_, maximal midexpiratory flow.

Each patient had a different total number of PFTs (median 6; IQR: 3–13). At each PFT, all patients underwent FVC, FEV_1_, FEV_1_/FVC ratio, and MMEF_25−75%_ tests. The median interval time between every two PFTs was 3 months (IQR: 1–9.5 months). As Figure 2 shows, over time, dramatic inter- and intraindividual variability in every PFT parameter occurred. However, even accounting for that variability, the FVC, and FEV_1_ improved in 11 children and only mildly decreased in one child. FVC and FEV_1_ increased by of 8.212%/year (95% CI: 6.531–9.894%; *p* < 0.0001) and 5.007%/year (95% CI: 3.463–6.552%; *p* < 0.0001), respectively (Table 2, Figure 2). The increase in FEV_1_ was not as significant as that in FVC, so a dramatic decrease occurred in the FEV_1_/FVC ratio, which represented an average reduction of 3.537%/year (95% CI: 1.984–5.09%; *p* < 0.0001) (Table 2, Figure 2). MMEF_25−75%_ improved in nine children and remained unchanged or declined slightly in three children, which resulted in an average improvement of 1.583% on a yearly basis (95% CI: 0.046–3.12%; *p* < 0.05) (Table 2, Figure 2).

Overall, FEV_1_, and MMEF_25−75%_ showed different degrees of improvement after inhaled bronchodilators at each PFT session for ten patients, and FEV_1_ measures demonstrated significance (>12%) with β_2_-bronchodilation in 56% of PFT sessions (Figure 2, data not shown).

## Discussion

This investigation followed PFT changes in pediatric patients with PIBO over time. The diagnosis of PIBO was in accordance with typical clinical and HRCT findings. In our study, all patients showed impaired pulmonary function with an obstructive pattern, but improvement in pulmonary function was also observed. We found that FVC and FEV_1_ greatly increased; however, the FEV_1_/FVC ratio declined significantly over time.

A total of 66.7% of the studied patients developed PIBO before 3 years of age, although two children in preschool and two of school age were diagnosed with PIBO. All participants were of Han Chinese ethnicity. In PIBO pathology, adenovirus and *M. pneumoniae* were the predominant microorganisms, occurring in 75% of the study subjects (nine out of 12 individuals), which was similar to the results reported in previous studies (3, 14, 15). In addition, measles virus was also a common cause. We also found that six patients required mechanical ventilation for the initial severe infection, and this is likely to be an independent risk factor associated with PIBO development (16).

In this group of patients, typical findings on HRCT chest scans were defined as a mosaic perfusion pattern because of patchy areas of hyperinflation and vascular attenuation, whereas air trapping was more apparent in expiration, along with bronchial wall thickening. We also identified atelectasis and bronchial dilation in some patients. One patient developed thoracic deformity because of right atelectasis.

PFTs are important for the diagnosis or follow-up of patients with PIBO. Pronounced reductions in FEV_1_, the FEV_1_/FVC ratio, and MMEF_25−75%_ were observed in the study, which are characteristic of obstructive airway disease, especially small airway disease. Our findings corroborate the conclusion that pediatric patients undergoing PIBO do share a common mode of serious pulmonary function impairment with the characteristic of marked airway obstruction (17–19). Meanwhile, the decrease in FVC seemed to be combined with restrictive dysfunction, but it was not real restricted when lung volumes measured by plethysmography were available.

In our patients, we observed that FVC, FEV_1_, and MMEF_25−75%_ were substantially improved over time. FVC increased more than FEV_1_, so the FEV_1_/FVC ratio significantly decreased. Although spirometry parameters increased, pulmonary function (PF) remained moderately impaired in childhood, especially in the small airways. The improvement in PF may be the result of conserved normal lung growth. The conception of neoalveolization from childhood to adolescence was postulated by Narayanan et al. (20). The decreased FEV_1_/FVC ratio is probably because of the unequal growth of the airways and lung parenchyma, indicative of “dysynaptic growth” of the lung. In other words, in terms of alveolar number, the catch-up growth following lung injury could be possible, but may not be as much to airway size (20, 21). For example, “those with PIBO are more likely to be volume responders than flow responders” (22). This has been observed in two other studies (7, 10) and was further confirmed by the present case series. Other research has drawn different conclusions, such as longitudinal data from 6 children showing unchanged abnormal lung function many years after treatment (8); a study including 11 patients with PIBO reported that pulmonary function declined with growth (9). This is possibly because we included a homogeneous group of younger children who had more time for alveolar development and relatively non-severe forms of the disease. Certainly the improvement in PF doesn't mean the regression of bronchopulmonary lesions, as showed by the not very significant changes of MMEF_25−75%_ (*p* = 0.048), indicating the involvement of small airways, and the reduction of number of children that showed bronchodilator response at final PFT. In addition, there is no widely acknowledged protocol for PIBO treatment. Therefore, it was difficult to demonstrate that the improvement in PF was associated with the treatment in our study.

At the initial PFTs, 10 (83.3%) pediatric PIBO patients demonstrated a significant bronchodilator response according to the ATS/ERS criteria; that is, FEV_1_ was significantly improved after inhalation of bronchodilators (on average 15.55%), but the number of children with positive results decreased during the follow-up, with seven (58.3%) at the final PFTs. Longitudinal assessment of the bronchodilator response over the follow-up period demonstrated that a positive response for FEV_1_ remained in over half of the PFT sessions (Figure 2). In children with PIBO, the most severe obstruction is in the small airways, so we observed higher β_2_ agonist responses in terms of the MMEF_25−75%_. However, MMEF_25−75%_ is usually highly variable in control groups and lacks a consistent standard; therefore, the variation is not easily interpreted (23). We did not find that either age at diagnosis or allergy-related factors exerted any dramatic effects on the level of the bronchodilator response. Despite theoretically, a bronchodilator response ought to be not observed in children undergoing fixed airway obstruction, as in PIBO, but the reversibility of airway obstruction in PIBO remains controversial. Mattiello et al. reported 72 children with PIBO in whom the bronchodilator response was significant in 42 patients (58.3%) (11). Chung and Jang (24) observed bronchial hyperresponsiveness in more than 40% of PIBO patients. Yoo et al. (25) found that some patients (78.6%) with PIBO showed hyperresponsiveness to methacholine. In Chile, Castro-Rodriguez et al. even found a dramatic bronchodilator response under observation in 94.4% of included children who had PIBO using impulse oscillometry (14).

Our study adds information that irreversible, fixed obstruction on PFTs does not seem to apply to all children with PIBO. The mechanisms underlying airway hyperreactivity in such patients remain unclear. This could be explained either by a predisposition (prior to the diagnosis) to PIBO in children with airway hyperreactivity (14); however, in young children, it is difficult to estimate bronchodilation in PFTs; or by acquired airway hyperreactivity in the disease development. There may be persistent airway hyperresponsiveness secondary to complex damage and bronchiolar repair, including airway chronic inflammation, fibrosis and other factors. However, the situation is different from asthma. One study (25) showed that children with asthma and those with PIBO have airway hyperreactivity to methacholine, but only those with asthma are hyperreactive to adenosine 5′-monophosphate, which indicates that underlying eosinophilic or atopic airway inflammation may be absent in PIBO patients. In addition, it was shown that the presence of non-specific bronchial hyperresponsiveness involved in the peripheral small airways in transplant patients is associated with the development of BO (26). Certainly, more attention should be given to asthma being overlooked or misattributed to PIBO, including asthma-PIBO overlap syndrome. However, in our patients, during the whole study period, mosaic perfusion and bronchial dilation persisted on HRCT, and the symptoms could not be completely resolved after systemic treatment. This condition is rarely or never seen in asthma; moreover, there were no positive findings in allergy tests, and there was no supporting evidence for asthma diagnoses. In addition, these differences might also be explained by dissimilar populations. However, it seemed that poor responses to bronchodilators increased as fibrosis progressed. Although, the PFT parameters did not achieve normality after the use of bronchodilators, these medications can help improve lung function in patients. Further investigations are needed to research the mechanisms of airway hyperresponsiveness and assess the benefits of the use of bronchodilators in PIBO patients with a significant bronchodilator response.

This study has several limitations. First, the sample size was small. Second, tracheal dimensions, which support the conception of “dysanaptic growth of lung and airways” directly, cannot be measured in our hospital. Furthermore, it was a retrospective study, the sample was not homogeneous because patients had difference in initial severity of disease, differences in follow up period and in age at which baseline and final PFT were performed.

In conclusion, the results of the current case series demonstrated that there was an obstructive pattern of pulmonary function impairment in children with PIBO. FVC, FEV_1_, and MMEF_25−75%_ all improved as the patients grew older, while the FEV_1_/FVC ratio decreased. This improvement may be due more to the development of lung parenchyma than to airway growth. The airway obstruction of some pediatric patients with PIBO can be alleviated by the application of β_2_ agonists. Future exploration should include a larger number of patients and longer follow-up periods, which is required for validation of the observations presented here.

## Data Availability Statement

The original contributions presented in the study are included in the article/supplementary material, further inquiries can be directed to the corresponding author/s.

## Ethics Statement

The studies involving human participants were reviewed and approved by the research ethics committees of First Hospital of Jilin University. Written informed consent to participate in this study was provided by the participants' legal guardian/next of kin.

## Author Contributions

XY undertook the follow-up of patients, data collection, analysis, and produced the manuscript. JW was responsible for the follow-up of patients and data collection. YL, LZ, and HC undertook data analysis and a literature review. LL was for the whole article and financial support as corresponding author. All authors contributed to the article and approved the submitted version.

## Conflict of Interest

The authors declare that the research was conducted in the absence of any commercial or financial relationships that could be construed as a potential conflict of interest.

## Abbreviations

PIBO: post-infectious bronchiolitis obliterans
PFTs: pulmonary function tests
FVC: forced vital capacity
FEV_1_: forced expiratory volume in 1 s
MMEF_25−75%_: maximal midexpiratory flow velocity 25–75%
HRCT: high resolution chest computed tomography.
